# Informal and formal care among persons with dementia immediately before nursing home admission

**DOI:** 10.1186/s12877-020-01703-8

**Published:** 2020-08-18

**Authors:** Arnt Egil Ydstebø, Jurate Šaltytė Benth, Sverre Bergh, Geir Selbæk, Corinna Vossius

**Affiliations:** 1 Centre for Age-related Medicine, Stavanger University Hospital, Stavanger, Norway; 2 Centre for Development of Institutional and Home Care Services Rogaland, Stavanger, Norway; 3 Research centre for Age-related Functional decline and Disease, Innlandet Hospital Trust, Postboks 68, N-2312 Ottestad, Norway; 4 Institute of Clinical Medicine, University of Oslo, Oslo, Norway; 5 Health Services Research Unit, Akershus University Hospital, Akershus, Norway; 6 Norwegian National Advisory Unit on Ageing and Health, Vestfold Hospital Trust, Tønsberg, Norway; 7 Institute of Health and Society, University of Oslo, Oslo, Norway

**Keywords:** Dementia, Informal care, Formal care, Resource use, Living situation, Social network

## Abstract

**Background:**

Dementia is a care intensive disease, especially in the later stages, implying in many cases a substantial carer burden. This study assesses the use of formal and informal care resources among persons with dementia during the last month before nursing home admission. It also describes main providers of informal care and assesses the extent of informal care rendered by the extended social network.

**Methods:**

In this cross-sectional study, we collected data about persons with dementia that were newly admitted to a nursing home in Norway. Information about the amount of formal and informal care during the last 4 weeks preceding nursing home admission was collected from the primary caregivers. Clinical data were collected by examining the patients, while sociodemographic data was collected from the patients’ files.

**Results:**

A total of 395 persons with dementia were included. The amount of informal care provided by the family caregiver was 141.9 h per month SD = 227.4. Co-resident patients received five times more informal care than non-co-residents. Informal care from the extended social network was provided to 212 patients (53.7%) with a mean of 5.6 (SD = 11.2) hours per month and represented 3.8% of the total informal care rendered to the patients. Formal care was provided to 52.7% of the patients with a mean of 18.0 (SD = 50.1) hours per month. Co-residency was significantly associated with more informal care, and the associations varied with respect to age, relation to the caregiver, and the caregiver’s working situation. Good/excellent general health was associated with less formal care.

**Conclusion:**

Persons with dementia on the verge of admission to a nursing home are mainly supported by the family caregiver, and the use of informal care is particularly high among co-residents. In order to delay nursing home admission, future research should explore the unrealized care potential in extended social networks, as well as the potential for increasing the number of recipients of formal care services.

## Background

The number of people living with dementia worldwide was estimated as 35.6 million in 2010, and the numbers are expected to double over the next 20 years [1]. Strategies are needed to secure sufficient support for people living with dementia as well as their informal and professional caregivers without financially overstraining health and welfare systems. Health economic evaluations show that nursing home placement is the most significant factor driving costs in dementia care and that the interval from diagnosis to institutionalization is about 30–40 months [2–5]. A considerable amount of informal care is provided in the patient’s home, and the burden of care is one of the main important factors associated with nursing home admission (NHA) [6–11]. Several studies report that a considerable amount of informal care is provided in the home environment from 30 up to 100 h per week in their respective populations [7, 10, 12–16]. Factors associated with increased informal care are dementia severity, severe neuropsychiatric symptoms, and increased functional impairment [7, 8, 12, 17–19]. The influence of co-residency between the primary caregiver and the person with dementia is of particular interest. Previous studies have found associations between co-residency and increased informal care, while more formal care was associated with persons with dementia living alone [12, 15, 17, 20].

Expecting an increasing number of persons with dementia, The Norwegian Ministry of Health published the first national Dementia Strategy in 2007 to better meet the needs of patients, family caregivers, and the health care system. This strategy was updated in 2015. One of the main goals of the Norwegian Dementia Strategy 2020 [21] is to enhance the support to the family caregivers and better the cooperation with voluntary services to empower persons with dementia to participate more actively in society and to live longer in their own homes. A study performed in eight European countries found that informal care from family and friends, in addition to the primary caregiver, were available to less than half of the carers in the study and suggested that provision of informal support to the carer may act as a protective factor for the perceived carer burden [14]. However, knowledge about the extent of informal care in Norway delivered during the period before NHA, and the providers of informal care, not only including primary caregivers but also the extended social network, is scarce. Better insight to the extent of care provided in patients’ homes, who provides the care, and what factors are related to the amount of care in the period before NHA may assist the development of new services aiming to prolong time to nursing home admission.

The aim of this study was to assess the resource use in formal and informal care among persons with dementia during the last month before NHA, and to assess clinical and sociodemographic factors associated with the use of care. We aimed as well as to describe the main providers of informal care, and to assess the amount of informal care rendered by the extended social network.

## Methods

### Setting

This is a cross-sectional study assessing baseline data in a sample drawn from the Resource Use and Disease Course in Dementia – Nursing Home (REDIC-NH) project.

### Study population

The study population was a sub-sample of participants in the- REDIC-NH project. The REDIC-NH study is a longitudinal observational study that includes newly admitted patients from 47 small and large nursing homes in four Norwegian counties and follows them from admission to the NH over a course of 5 years or until death [22]. Patients older than 65 years, or younger than 65 years but with established dementia, were included. In addition, the expected stay in the NH had to be more than 4 weeks. Patients with a life expectancy shorter than 6 weeks were not eligible. The study included a convenience sample of 695 persons, and recruitment took place between January 2012 and August 2014.

To increase homogeneity and describe the resource use in a dementia population exclusively, patients without dementia, or not permanently admitted to NH where excluded from the present study, as were participants without complete Resource Utilization in Dementia (RUD) questionnaire.

To be admitted to a long-term NH stay in Norway, the person must apply to the municipality. The application is evaluated based on a needs’ assessment, and available places are allocated based on urgency. If there are no available places, the applicant usually is placed on a waiting list, with a waiting period from a few days up to several weeks.

### Ethical considerations

The patients’ capacity to consent was assessed by the nursing home staff, including a physician. Written informed consent was obtained from patients with the capacity to consent or from the family caregivers on behalf of the patients in cases of reduced capacity to consent. The study was approved by the Regional Committee for Medical and Health Research Ethics (2011/1738).

### Data collection

Data were collected by healthcare workers at the nursing home, under the supervision of 10 research nurses. The research nurses completed 5 days of training prior to the start of the study, and the data collectors completed 2 days of training. Data were collected through cognitive and physical tests and structured interviews with the patients, their family caregivers defined as a next of kin who looked after the patient at least once a week, and the health workers.

### Measures

Demographic data included the patients’ age and gender and were collected by reviewing the patients’ files kept at the NHs. A diagnosis of dementia according to the ICD-criteria [23] was independently established by two of the authors (SB and GS) based on all available information about the patients. Both SB and GS are specialists in psychiatry and experienced in geriatric psychiatry and research. If no consensus was reached, a third psychiatrist was consulted.

The clinical measures dementia severity and severity of physical health were obtained using the following instruments: The General Medical Health Rating (GMHR) [24], a four-category, reliable, and valid global bedside assessment tool for rating the severity of physical health. The score is based on an overall assessment by health care workers. The Clinical Dementia Rating Scale (CDR) assesses the severity of dementia as no dementia, possible dementia, and mild, moderate, or severe dementia. CDR comprises six items (memory, orientation, judgement and problem solving, community affairs, home and hobbies, and personal care). A score is calculated according to an algorithm where the memory item is given more weight. For statistical purposes, we calculated the CDR sum of boxes (CDR-SOB), which offers an extended range of values and is calculated by adding the item scores (range 0–18). Higher scores indicate more severe dementia [25].

The extent of formal care and the extent and providers of informal care during the last 4 weeks preceding NHA were recorded by the RUD questionnaire, that is answered by the primary caregiver and includes the following information about the primary caregiver [26]: Age, gender, relation to the patient, co-residential status, work status, hours worked last month, and lost work hours due to care tasks in the last month. Information about the extended social network included: Relation to patient and hours of provided informal care last month. The extent of informal care provided by the family caregiver last month was recorded in regard to the following three aspects: 1) the time used to help the patients with personal activities of daily living (PADL), 2) the time used on instrumental ADL (IADL), 3) and the time used on supervision, like helping the patient with orientation or preventing behavior that is distressing to the patient. We calculated the total informal care time by summarizing the amounts of time for these three types of care. If this sum exceeded 24 h per day, a total informal care time of 24 h per day was set. Formal care was equalled to the time provided by professional home care services, while services like home help, meal delivery, day care centres, or respite care were not included due to insufficient data.

### Statistical analysis

Data are described by the means and standard deviations (SD) or frequencies and percentages. Differences between groups were assessed by Independent Samples t-test for continuous variables and the χ^2^-test for categorical variables. Associations between predefined covariates (gender, age, caregiver gender, caregiver relation to patient, caregiver in paid work, co-residency with family caregiver, GMHR, and CDR) and the three outcome variables (informal care by the primary caregiver, informal care by the extended social network and formal care) were assessed by estimating bivariate and multiple linear mixed models. Random effects for nursing homes were included in the models to adjust the estimates for possible within-nursing-home correlations. Stratification by living with or without a caregiver was performed by including interactions between the dichotomous variable (co-residency with or without caregiver) and all covariates. Interactions with *p* < 0.1 were kept in the model. Missing values in variables co-resident, carer relation, and carer in work were imputed by logical rules whenever possible. The analyses were performed using IBM SPSS Statistics for Windows version 25.0 (Armonk, NY: IBM Corp.) and SAS v 9.4. Results with *p*-values less than 0.05 were considered statistically significant.

## Results

### Study population

The REDIC-NH cohort consists of 696 patients, among which 445 had dementia and were permanently admitted to a nursing home. However, 50 patients had to be excluded due to missing or incomplete RUD questionnaires. Thus, the study included 395 patients with a mean age of 84.4 (SD = 7.5) years, and 265 (67.1%) patients were female. According to the CDR, 277 (73.9%) had moderate or severe dementia. There were no differences regarding demographic or clinical characteristics between patients who completed the RUD questionnaire and those who did not.

The extent of formal and informal care is presented in Table 1.

**Table 1:** Formal and informal care during the last month before NHA (*N* = 395)

Variable	
Formal care	
Recipients, n (%)	208 (52.7)
Mean hours of formal care, recipients of formal care, last month (SD)	34.2 (64.9)
Mean hours of formal care, whole sample last month (SD)	18.0 (50.1)
Informal care by the primary carer, mean hours last month (SD)	
PADL	74.8 (170.5)
IADL	65.9 (142.1)
Supervision	55.9 (158.9)
Total informal care by the primary carer	141.9 (227.4)
Informal care by wider social network, mean hours last month (SD)	
Family	3.8 (10.2)
Relatives	0.4 (3.0)
Friends	0.1 (0.8)
Neighbours	0.2 (1.0)
Others	0.9 (4.0)
Total informal care by the extended social network	5.6 (11.2)

*NHA Nursing Home Admission, SD Standard Deviation*

### The extent of informal care

The mean care time provided by the family caregiver was 141.9 (SD = 227.4) hours per month, while the total contribution of the patients’ extended social network, including family members, was 5.6 (SD = 11.2) hours per month.

### The extent of formal care

Formal care was provided to 208 (52.7%) of the patients with a mean of 34.2 (SD = 64.9) hours per month among those receiving formal care. For the whole sample, the mean amount of formal care per month was 18.0 (SD = 50.1) hours.

### Informal care – characteristics of the family caregiver

Characteristics of the family caregivers and the extended social networks are presented in Table 2. Of the 395 patients, 379 (95.9%) had a family caregiver, of whom 228 (60.2%) were females, 255 (67.3%) were the patients’ children, while 81 (21.4%) were spouses. The mean age was 57.4 (SD = 8.9) years for family caregivers who were the patients’ children and 77.7 (SD = 7.6) years for family caregivers who were the patients’ spouses. Co-resident caregivers accounted for 105 (26.6%) of the sample. A total of 194 (54.2%) caregivers were doing paid work, and they worked 34.9 (SD = 10.3) hours per week. Of the caregivers in paid work, 60 (30.8%) reported a mean loss of 11.2 (SD = 9.6) working hours per week due to care tasks.

**Table 2:** Description of the family caregivers (*n* = 379) and the patient’s extended social network

Family caregiver’s relation to patient, n (%)	
- spouse	81 (21.4)
- child	255 (67.3)
- others	43 (11.3)
Age, mean (SD)	
- spouse-carers	77.7 (7.6)
- child-carers	57.4 (8.9)
Gender, n (%) *female*	228 (60.2)
Co-resident, n (%) *yes*	105 (26.6)
Employed, n (%) *yes*	194 (54.2)
Hours worked per week if in paid work, mean (SD)	34.9 (10.3)
Carers that lost working hours due to care tasks, n (%)	60 (30.8)
Mean hours lost per week, if reporting lost hours (SD)	11.2 (9.6)
Care benefit as part of paid work, n (%) *yes*	8 (4.0)
Mean hours care benefit per week, if receiving care benefit, mean (SD)	7.6 (5.6)
Number of additional care providers, n (%)	
- 0	183 (46.3)
- 1	172 (43.5)
- 2	28 (7.1)
- 3	12 (3.7)
Additional care providers’ relation to patients, n (%)	
- family	154 (72.6)
- relatives	29 (13.7)
- friends	15 (7.1)
- neighbours	22 (10.4)
- others	44 (20.8)

*SD Standard Deviation*

### Informal care - characteristics of the extended social network

In our study sample, 212 (53.7%) patients received support from at least one member of their social network, whereas 183 (46.3%) had no additional carers beside the family caregiver. Of the 212 patients that received help from their extended social network, 154 (72.6%) received help from family members, while 29 (13.7%) received help from more distant relatives, 15 (7.1%) received help from friends, 22 (10.4%) received help from neighbours, and 44 (20.8%) received help from others (Table 2).

### The impact of sociodemographic and clinical factors on the use of care

**Fig. 1:**
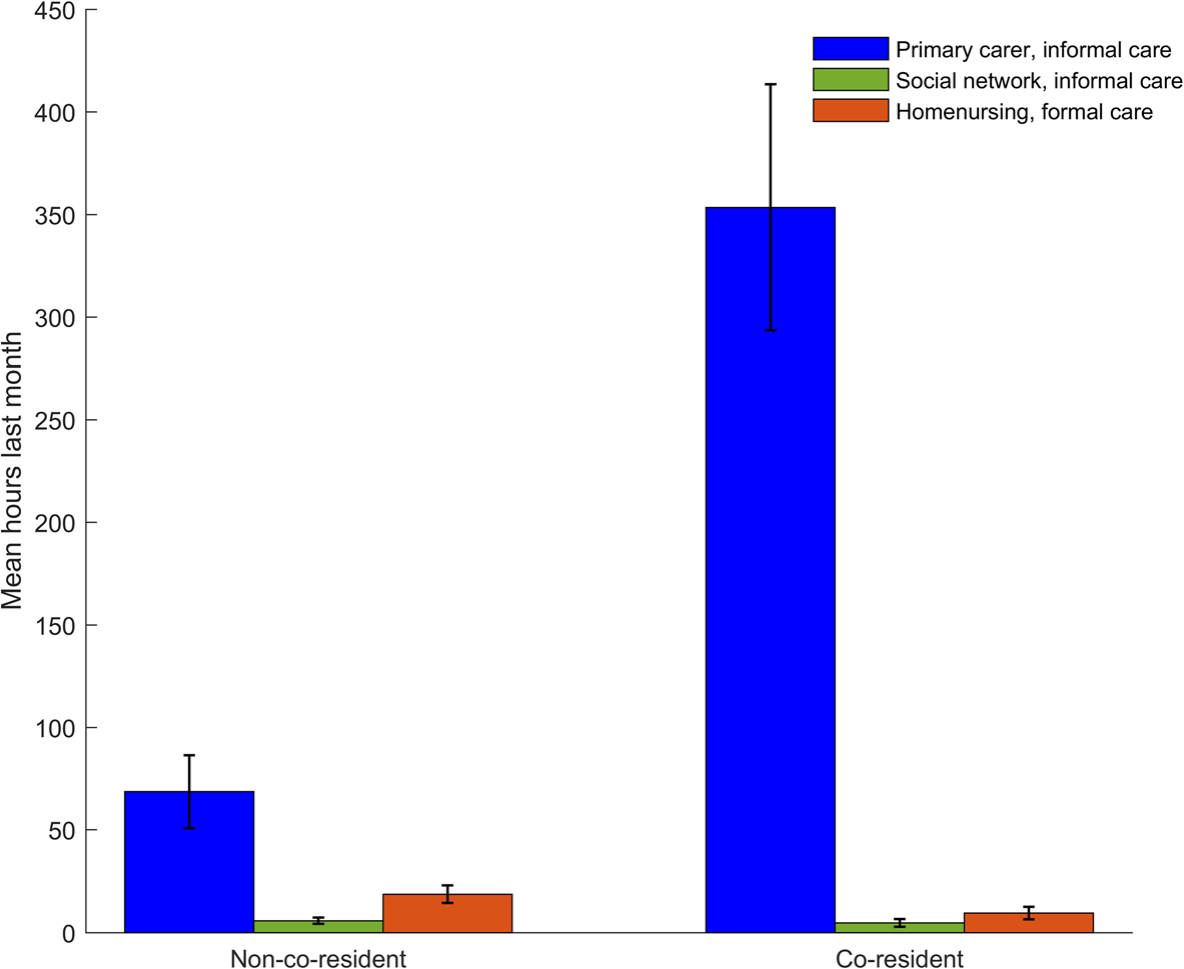
Time used to care for patients during the last month before nursing home admission

Male patients received more informal care than female patients (187.1 versus 120.3 h per month, *p* = 0.016). There was no statistical difference between genders regarding formal care. Co-resident patients received more informal care (343.1 versus 67.4 h per month, *p* < 0.001) and less formal care than patients living alone (9.1 versus 21.2 h per month, *p* = 0.001), while there was no statistical difference in the extent of informal care by the extended social network between co-residents and non-co-residents (Fig. 1). The ratios of informal to formal care were 37.7:1 for co-resident patients and 3.2:1 for patients living alone. Family caregivers who did paid work provided less informal care than those who were not working (74.2 versus 228.7 h per month, p < 0.001). There were no statistical differences in formal care between patients with working and non-working family caregivers.

According to the bivariate linear mixed models presented in Table 3, more hours of *informal care by the family caregiver* was significantly associated with the patient being of lower age, having male gender, having a family caregiver not doing paid work, having a spouse as a family caregiver as opposed to a child, having more severe dementia, and having a co-resident caregiver. Having a family caregiver doing paid work was associated with more *informal care from the social network*. Having a spouse as the family caregiver as opposed to a child, good/excellent GMHR, and co-residency were associated with less *formal care*.

**Table 3:** Bivariate and multiple models of formal and informal care

Independent variables	Dependent variables					
	Informal care by primary carer (*N* = 344)		Informal care, social network (*N* = 316)		Formal care (*N* = 356)	
	Regr.coeff. (95% CI / SE)	*p*-value	Regr.coeff. (95% CI)	*p*-value	Regr.coeff. (95% CI)	*p*-value
BIVARIATE MODELS						
Gender, male	76.9 (25.6; 128.2)	**0.003**	1.1 (−1.6; 3.9)	0.428	1.4 (−6.8; 9.6)	0.738
Age	−6.9 (− 10.1; −3.8)	**< 0.001**	−0.1 (−0.3; 0.0)	0.120	0.1 (− 0.5; 0.6)	0.841
Carer gender, male	−21.5 (− 71.1; 28.2)	0.396	− 0.1 (− 2.7; 2.5)	0.925	− 0.36 (−8.20; 7.5)	0.929
Carer relation						
Spouse	302.4 (250.8; 353.9)	**< 0.001**	−3.0 (− 6.1; 0.2)	0.062	−9.7 (− 19.3; − 0.1)	**0.047**
Children	0		0		0	
Others	41.2 (−25.7; 108.1)	0.227	−2.6 (−7.0; 1.7)	0.235	3.7 (−8.5; 16.0)	0.549
Carer in work, yes	− 155.6 (−201.6; − 109.6)	**< 0.001**	3.7 (1.1; 6.2)	**0.005**	4.7 (−3.0; 12.4)	0.230
GMHR, poor/fair	6.1 (−42.5; 54.8)	0.804	−0.9 (−3.5; 1.7)	0.480	8.8 (1.2; 16.4)	**0.024**
CDR-SB	8.5 (1.5; 15.6)	**0.017**	0.2 (−0.2; 0.5)	0.433	−0.2 (−1.3; 0.9)	0.699
Co-resident	275.6 (229.8; 321.4)	**< 0.001**	−1.1 (−3.9; 1.8)	0.453	−11.6 (−20.1; − 3.1)	**0.008**
MULTIPLE MODELS						
Gender, male	21.5 (26.6)	0.419	1.8 (−1.1; 4.8)	0.217	2.6 (−6.1; 11.2)	0.565
Age	−2.0 (1.7)	0.229	−0.2 (−0.4; 0.03)	0.094	−0.1 (− 0.6; 0.5)	0.790
Carer gender, male	−45.0 (− 89.0; −1.0)	**0.045**	0.5 (− 2.2; 3.1)	0.743	1.2 (−6.9; 9.19)	0.774
Carer relation						
Spouse	298.8 (74.5)	**< 0.001**	−4.5 (−9.8; 0.75)	0.093	−1.9 (−17.6; 13.9)	0.813
Children	0		0		0	
Others	10.6 (35.1)	0.763	−1.9 (−6.3; 2.6)	0.411	3.8 (−8.8; 16.5)	0.551
Carer in work, yes	−29.3 (26.7)	0.273	2.5 (−0.6; 5.6)	0.110	1.3 (−8.0; 10.47)	0.790
GMHR, poor/fair	14.1 (−25.2; 53.5)	0.481	−0.9 (−3.4; 1.7)	0.518	8.5 (0.8; 16.2)	**0.030**
CDR-SB	−0.3 (−6.1; 5.5)	0.921	0.2 (−0.2; 0.5)	0.411	0.03 (−1.1; 1.2)	0.964
Co-resident	1077.4 (314.7)	**0.001**	1.81 (−2.3; 5.9)	0.383	−10.0 (− 22.3; 2.3)	0.110
Gender x Co-resident	95.09 (52.70)	0.072				
Age x Co-resident	−8.74 (3.34)	**0.009**				
Carer relation x Co-resident						
Spouse	−321.03 (98.72)	**0.001**				
1 Children	0					
Others	127.97 (107.31)	0.234				
Carer in work x	−154.95 (66.30)	**0.020**				
Co-resident						

*CI Confidence Interval, SE Standard Error, GMHR General Medical Health Rate, CDR-SB Clinical Dementia Rating Sum of Boxes*

In the multiple model, female gender of caregiver was significantly associated with more hours of informal care by the family caregiver. Several interactions with co-residency were present in the model. Co-resident patients had significantly more hours of informal care than non-co-resident patients with differences varying between strata. There were no differences between male and female patients. Differences in provided informal care between spouses and children caregivers were significantly larger among non-co-resident patients than co-resident patients, Differences in received informal care from working and non-working caregivers were significantly higher among co-resident patients than non-co-resident patients. Higher age was associated with fewer hours of informal care, and there was a significantly stronger reduction among co-resident patients than non-co-resident patients. Less formal care was only associated with good/excellent GMHR.

## Discussion

This study assessed the use of formal and informal care among home-dwelling persons with dementia during the last month before permanent admission to a nursing home. For the two-thirds of the patients living alone, their closest caregiver was mainly one of their children, while for the patients living in co-residency, the caregiver was most frequently the spouse. Half of the sample received formal care in the form of home care services. The extent of informal care was substantially larger than the extent of formal care, and the majority of the informal care was delivered by the family caregiver, while the extended social network only contributed 3.8%. Half of the primary caregivers even reported to have no additional help at all, including the majority of spouses living with the patient. Only a small portion of 10% received care from two or more persons in their extended social network; however, the low hourly contribution suggests that the help was somewhat sporadic.

Care contributions from the extended social network have seldom been investigated or discussed in previous studies. In the US, The National Study of Caregiving found that 73–78% of caregivers to persons with dementia had additional helpers [27]. In Norway, the REDIC project found that 50 to 70% of caregivers had additional helpers, however, our findings show that the hourly contribution was low. We can only suggest possible reasons for the low contribution. Norway has high employment rates among both genders, possibly reducing the opportunity to support relatives [28]. Another explanation could be the increasing urbanization causing longer geographical distance between relatives [29]. The massive expansion of the public care services during the last 50 years might also lead to a perception that the provision of care to elderly relatives is a state- rather than a family-responsibility [30]. As more then 95% of all informal care was rendered by the main caregiver, there seems to exist unexhausted resources in involving the extended social network in order to relief the burden experienced by primary caregivers. However, the research community has yet to explore how to access these resources and the barriers that might exist in both rendering care but as well accepting it.

We found that the family caregiver provided a mean of 142 h per month of informal care. In contrast, a health economic analysis performed on several cohorts of persons with dementia concluded that a mean of 60 to 85 h per months of informal help were rendered at the point of diagnosis, thus, indicating a considerable increase in the need for care in the period leading up to NHA [31]. A previous study conducted in eight European countries with a cohort similar to the REDIC cohort reported 360 h of informal care per month, while studies observing cohorts with younger patients in earlier stages of dementia reported a range of 82 to 160 h per month [12, 14–16]. These differences might be owed to varying shares of co-residing family caregivers. In addition, cultural differences regarding the experienced obligation to care for elder family members as well as the accessibility and costs of formal care might impact the extent of informal care.

Co-residency was a main predictor for the extent of informal care rendered. This might be an indication of “supply creates its own demand”, as co-resident caregivers (the spouse in most cases) might be more involved than needed when assisting the patients with daily tasks and supervision [32]. It could as well lead to an overestimation of care time, as it might be difficult to distinguish shared household activities from care activities. On the other hand, more severe dementia was associated with more informal care, suggesting that the amount of informal care is adjusted to the severity of dementia.

Only about half of the sample received formal care and our finding of 4.5 h of formal care per week is considerably lower than the findings in a comparable study that reported 7.5 h [15]. Another analysis of care resources to Norwegian home-dwelling persons with and without dementia found that 3.2 h of formal care was provided to a sub-population with dementia [31]. Thus, indicating a progressive increase in formal care provision. In recent years Norwegian Dementia Strategies have asked for more differentiated care services and a more individualized approach toward persons with dementia and their family caregivers. Consequently, we would expect a higher amount of informal care time and dementia-specific clinical measures to be associated with the extent of formal care, but we found that only somatic health was related to it. A possible explanation could be that Norwegian home care services mainly cover help with tasks related to ADL dependencies and, to a lesser degree, with tasks related to IADL dependencies and supervision of the patients [33].

Our finding that co-resident patients received less formal care and more informal care than patients living alone is consistent with previous studies [12, 15–17]. It indicates a substitutive rather than complimentary relationship between formal and informal care use and are in line with a recently published study in six Western-European countries [34]. This might as well apply to the contributions from the extended social network as more support was provided from the extended social network in cases with non-co-residency and when the primary carer was holding a job.

The low use of formal care substituted by a high use of informal care might be due to a lack of perceived capacity or skill in the primary care services to offer specialized and individualized dementia care, especially care and support directed towards co-resident caregivers. A cross-European study found that the formal services available to persons with dementia were non-specific and not tailored to the patient group or the specific individual’s needs [35]. Other identified barriers to the use of formal care are that the family caregivers do not consider the need for the care, them or the patient having negative attitudes and beliefs towards formal care, low awareness of services available, poor accessibility to services, or high costs [36–38]. Another possible barrier to formal care derives from a Canadian study were case managers seemed to purposely exhaust family resources before making formal home care services available [39]. Increasing the number of recipients of formal care or increasing the hours of care delivered to the respective recipients might contribute to relief the burden of primary caregivers and thus to delay NHA [5, 40].

### Limitations and strengths

The strengths of this study include a large sample of nearly 400 persons with dementia who were assessed for informal and formal care used during the last month before NHA. Standardized interviews were carried out by adequately trained and supervised healthcare workers, thus securing high-quality data. Private entities rarely provide health care service in the municipalities in Norway. Thus, the municipalities are almost exclusively responsible for the provision of care services and provide a homogenous environment for health service research with similar criteria for NHA.

A major limitation is that our sample might not be representative of the general population of persons with dementia in this stage in Norway as only patients that completed the BL examination were included, and the mean time from admission to BL was 10.5 weeks [22]. As a confounding factor we might thus have excluded patients who were eligible for the study but who died shortly after admission to a nursing home, or eligible persons that did not have a family caregiver. Furthermore, the physical and cognitive tests were first performed at the BL examination and could be sensitive to changes during this time period or due to the event of admission. However, the GMHR and CDR have shown to be stable over time [41–43]. Moreover, caregiver-reported data from the RUD questionnaire may have yielded inaccuracies in the extent of formal and informal care.

We equated formal care with home care services without taking into account other forms of services, such as meal delivery, day care centres, or respite care due to insufficient or lacking data. This might have resulted in an underestimation of the extent of formal care. However, we consider home care services as the most relevant type of formal care in Norway, and as well when comparing different health care systems.

This study was performed in Norway, and the findings might not be transferrable to other countries with different health systems. In Scandinavia, public health services provide the majority of care, and it is rendered free of charge for relatively small fees to the patients. Thus, the extent of formal care, the point of time for NHA and the extent of informal care might differ substantially in other health care systems and cultural settings.

## Conclusion

We found that persons with dementia on the verge of admission to a nursing home are mostly supported by informal care provided by one primary carer, while the amount of care provided by the patients’ extended social network and the provision of formal care was low. Future research should explore the unrealized care potential in the extended social networks and the possibilities in more diverse formal care services directed to persons with dementia and their caregivers.

## Data Availability

The datasets generated and/or analysed during the current study are available for researchers in cooperation with the data owner, the Research Centre for Age-related Functional decline and Disease – Innlandet Hospital Trust. Information is available on the following page link: https://sykehuset-innlandet.no/avdelinger/alderspsykiatrisk-forskningssenter

## Abbreviations

NHA: Nursing home admission
REDIC-NH: Resource Use and Disease Course in Dementia-Nursing Home
RUD: Resource utilization in dementia
P-ADL: Personal activity of daily living
IADL: Instrumental activity of daily living
GMHR: General medical health rating
CDR: Clinical dementia rating
SD: Standard deviation
